# Supplementary material for: Tutorial: Guidelines for Single-Cell RT-qPCR
Source: Cells. 2021 Sep 30;10(10):2607. doi: 10.3390/cells10102607 (PMC8534298; doi:10.3390/cells10102607)
Supplement: Supplementary file 1 [file cells-10-02607-s001.zip › cells-1358813-supplementary.pdf]

**Supplementary material**

Tutorial: guidelines for the single-cell RT-qPCR

Here, we provide a step-by-step RT-qPCR protocol for the analysis of individual cells or small bulk samples. The protocol was set-up based on our best knowledge, underwent careful optimization and its validity and robustness was verified in numerous publications, mainly in the field of neurobiology [1–10]. The protocol was routinely updated to reflect recent development in the field, significantly influenced by our own research [11–19]. On the purpose, we do not describe the process of sample processing and single-cell collection, as these steps are dependent on the sample characteristics and cannot be generalized. We provide a list of reagents as well. If not specified otherwise, the reagents may be substituted based on the researcher's experience or laboratory convention. Although the presented protocol is well-suited for various experimental scenarios, we encourage users to adjust it based on the experiment-specific requirements or new advances in the field of the scRT-qPCR analysis.

## 1. Reverse Transcription

### 1.1. Samples and Reagents

- Samples stored at –80 °C in RNase-/DNase-free tubes or plates
- Maxima H Minus Reverse Transcriptase (ThermoFisher Scientific, Waltham, MA USA), cat. no. EP0752
- RNaseOUT™ Recombinant Ribonuclease Inhibitor (ThermoFisher Scientific, Waltham, MA USA), cat. no. 10777019
- Equimolar mix of oligo(dT)$_{15}$ primers and random hexamers (N$_6$) (50 μM each)
- Mix of dNTPs (10 mM each)

TATAA Universal RNA Spike I or II (TATAA Biocenter, Göteborg, Sweden), cat. RS25SI or II

Notes:

- We recommend testing storage vessels at –80 °C to document issues with evaporation or cross-contamination.
- Maxima H Minus Reverse Transcriptase has been recently benchmarked as top-performing RTase [11,20]. For higher cost per reaction, SuperScript™ IV Reverse Transcriptase (ThermoFisher Scientific, Waltham, MA USA, cat. no. 18090010) could be used as an alternative.
- For specific applications, consider separate usage of oligo(dT) and random hexamers. Concentration adjustments are feasible. For the details, see chapter ***Priming Strategy*** in the main text.
- RNA spike-in may be *in-house* designed using artificial sequence or sequence from other non-related species and prepared in bulk using a kit for in vitro transcription.
- RNA spike-in may be added already into the lysis buffer during cell collection process. Its signal will then reflect not only the RT efficiency, but also the quality of storage conditions. Adding multiple various spike-ins at different workflow stages is recommended.
- Primers, dNTPs and spike-ins should be prepared and stored in aliquots for single use.

### 1.2. Protocol

Follow-up steps describe the protocol when single cells were collected into 5 μl of lysis buffer (e.g., nuclease-free-water supplemented with bovine serum albumin [17]). Volume adjustment is possible, but keep in mind the dilution effect has on the overall scRT-qPCR sensitivity.

*Timing: preparation and pipetting of 15 min; thermal protocol of 50 min.*

a. Thaw, vortex and spin all the reaction components except the enzyme before use. Thaw samples on ice and also spin briefly. All the pipetting steps are performed on ice.

b. Reverse transcription is carried out in two steps (RT1 and RT2), starting with the addition of primers, dNTPs and spike-in to the sample. If more than one RT reaction is performed, prepare the mastermix including minimal 10% surplus. Mix the following components in an RNase-/DNase-free tube:

| RT1 | Volume Per One RT Reaction (µl) | Stock Concentration | Final Concentration |
|---|---|---|---|
| Nuclease-free water | 0.5 | – | – |
| RT primer pool | 0.5 | 50 µM | 2.5 uM |
| dNTPs | 0.5 | 10 mM | 0.5 mM |
| Spike-in* | 0.5 | – | – |
| Sample | 5.0 | – | – |

** Concentration of the RNA spike-in needs to be adjusted to mimic single-cell/small bulk concentration, but simultaneously maintains low variability between the measurements. Typically, RNA spike-in concentration that reports Cq values around 30 cycles represent a good compromise.*

c. Add 2 µl of RT1 mastermix to individual samples, briefly spin the contents and incubate:

| Temperature | Time |
|---|---|
| 65 °C | 5 min |
| 20 °C | 20 s |
| 4 °C | hold |

d. During the incubation prepare mastermix for the second step (RT2). If performing more than one RT reaction, prepare the mastermix including minimal 10% surplus. Mix the following components in an RNase-/DNase-free tube:

| RT2 | Volume Per One RT Reaction (µl) | Stock Concentration | Final Concentration |
|---|---|---|---|
| Buffer | 2.0 | 5× | 1× |
| RNase OUT* | 0.5 | 40 U/µl | 2 U/µl |
| Maxima H* | 0.5 | 200 U/µl | 10 U/µl |
| Sample + RT1 mastermix | 7.0 | - | - |

** Reduced concentration of Maxima H and RNase OUT (up to 50%) may be considered for routine and cost-effective single-cell measurements without negative effect on scRT-qPCR performance.*

e. Add 3 µl of RT2 mastermix to individual samples, briefly spin the contents and incubate:

| Temperature | Time |
|---|---|
| 30 °C | 10 min |
| 50 °C* | 30 min |
| 85 °C | 5 min |
| 4 °C | hold |

** For GC-rich RNA, the temperature can be increased to 65 °C.*

f. Place the samples on ice for an approximately one min to condensate the contents on the well bottom.

g. Leave the cDNA undiluted. For short-term storage, keep cDNA at 4 °C (up to 24 h). For long-term storage, keep the cDNA at –20 °C.

## 2. Preamplification

### 2.1. Reagents

- PreAMP assay pool prepared as 250 nM equimolar mixture of assays of interest
- IQ Supermix buffer (Bio-Rad, Hercules, California USA), cat. no. 1708860

Notes:

- PreAMP assay pool needs to be prepared with an extra care avoiding contamination and pipetting errors, especially when large pools are prepared. Think through the working layout in advance, keeping in mind the requirements on the working space and good pipetting practice.
- Prepare the preAMP assay pool by combining assays of interest in an RNase/DNase-free microtube. Store at –20 °C in aliquots to prevent freeze-thawing.
- PreAMP assay pool should be composed of well-optimized assays. Details on the assay validation procedure are listed in the 3.3 qPCR Assay Validation.
- Addition of control assays in the preAMP assay pool is highly recommended, e.g., spike-in assays and ValidPrime® (TATAA Biocenter, Göteborg, Sweden, cat. no. A106P25) for genomic background. Add appropriate control samples into the sample list as well (non-template control, positive control, gDNA etc.).
- The application of other PCR mastermixes is possible as well.
- Use of 8-channel repeat pipette can drastically reduce hands-on time.
- Test for sample quality control may be performed before samples are preamplified. For more details, please refer to 3.4 Sample Quality Control.

### 2.2. Protocol

Follow-up protocol outlines the protocol for targeted preamplification of single cells performed in 40-μl reactions (4 μl of undiluted cDNA representing 40% of cell's transcriptome is added in the reaction, keeping mandatory 10× cDNA dilution to prevent the inhibitory effect of RT reagents). For single cells, lower reaction volume may lead to loss of sensitivity for transcripts presented in few copies per cell. For small bulk samples, cost-efficient reaction volume (10 or 20 μl) may be used, as even low expressed transcripts are supposed to be presented in detectable concentration (our unpublished data). For the single-cell samples, we recommend using total volume 40-μl reactions.

*Timing: preparation and pipetting of 25 min (preAMP assay pool ready)—65 min (preAMP assay pool not ready); thermal protocol of 120 min.*

a. Thaw, vortex and spin all the reaction components before use. Thaw samples on ice and spin briefly. All the pipetting steps are performed on ice.
b. Prepare preAMP mastermix. If more than one preAMP reaction is performed, prepare the mastermix in 10% surplus. Mix the following components in a DNase-free tube:

| preAMP | Volume Per One preAMP Reaction (μl) | Stock Concentration | Final Concentration |
|---|---|---|---|
| IQ Supermix buffer | 20.0 | 2× | 1× |
| Nuclease-free water | 12.0 | – | – |
| preAMP assay pool | 4.0 | 250 nM (equimolar) | 25 nM (equimolar) |
| undiluted cDNA | 4.0 | – | – |

c. Briefly vortex and spin the preAMP mastermix and pipette 36 μl per reaction well.

d. Add 4 µl of undiluted cDNA sample to the individual wells, briefly spin and incubate:

| Temperature | Time | |
|---|---|---|
| 95 °C | 3 min | |
| 95 °C | 20 s | |
| 57 °C* | 4 min | 18 cycles |
| 72 °C | 20 s | |
| 4 °C | hold | |

** Adjust according to mean Tm of the preAMP assay pool.*

e. Immediately after the reaction, place the samples on ice and 4× dilute the reaction contents with cold nuclease-free water (i.e., 40 µl preAMP + 120 µl NFW). Open the reaction vessels with care to avoid contamination (preferably in the post-PCR room).

f. Store at –20 °C.

### 2.3 preAMP Assay Pool Validation—The ΔCq Method

PreAMP assay pool validation is performed as quality control of the amplification reproducibility. Each new preAMP assay pool should be subjected to the performance validation before enrollment in an experiment.

*Timing: Reverse Transcription of ~70 min in total; Preamplification of ~140 min in total; low-throughput qPCR of ~120 min in total.*

a. For samples, use three replicates of cDNA prepared from bulk-extracted RNA (~250 ng per RT) by reverse transcription protocol of choice (1. Reverse Transcription). Dilute prepared cDNA 4× in nuclease-free water (i.e., 10 µl cDNA + 30 µl nuclease-free water). To screen for the effect of genomic background, include gDNA sample (~1 ng per reaction to mimic low level of gDNA contamination, equivalent of hundreds of genomes). Specificity of the reaction is monitored using the non-template sample (nuclease-free water).

b. Setup the preamplification reaction according to 2.2. Preamplification Protocol, using the preAMP assay pool to be validated. Optionally, preamplify the cDNA in duplicates. Dilute the amplified samples 50× in nuclease-free water in post-PCR room.

c. Preamplified cDNA is subjected to conventional low-throughput qPCR. Prepare the qPCR reactions according to 3.1. Low-Throughput qPCR Protocol. For input, use 50× diluted amplified samples and 50× diluted non-preamplified samples (the cDNA used for input in preAMP). Run all the samples in qPCR duplicates.

d. Assays profiled in the qPCR measurement should include representative assays (cell-type markers, genes of interest) and spike-in. Moreover, the assays should cover range of high–medium–low expressed transcripts. At minimum eight assays should be profiled. Measure in qPCR duplicates.

e. The preAMP assay pool performance is reflected in the variance of the difference between the preamplified and non-preamplified cDNA ($SD_{\Delta Cq}$). Firstly, ΔCq is calculated separately for each of the cDNA replicates. The variance is then calculated as standard deviation of the individual ΔCqs. ΔCq is calculated for each assay separately. preAMP assay pool scoring $SD_{\Delta Cq} < 0.5$ for each of the validation assays is considered reliable.

| | Sample | Cq | qPCR Mean | qPCR SD | preAMP Mean | preAMP SD | non-preAMP - preAMP ΔCq | non-preAMP - preAMP SD (ΔCq) | preAMP mix Mean | preAMP mix SD |
|---|---|---|---|---|---|---|---|---|---|---|
| Glul | A 1.1 | 15.37 | 15.36 | 0.01 | 15.30 | 0.08 | 10.55 | 0.09 | 10.37 | 0.16 |
| | A 1.1 | 15.35 | | | | | | | | |
| | A 1.2 | 15.26 | 15.24 | 0.03 | | | | | | |
| | A 1.2 | 15.22 | | | | | | | | |
| | 1.1 | 25.88 | 25.85 | 0.03 | | | | | | |
| | 1.1 | 25.83 | | | | | | | | |
| | A 2.1 | 15.11 | 15.15 | 0.06 | 15.16 | 0.02 | 10.29 | 0.04 | | |
| | A 2.1 | 15.19 | | | | | | | | |
| | A 2.2 | 15.23 | 15.18 | 0.07 | | | | | | |
| | A 2.2 | 15.13 | | | | | | | | |
| | 2.1 | 25.43 | 25.46 | 0.04 | | | | | | |
| | 2.1 | 25.48 | | | | | | | | |
| | A 3.1 | 15.27 | 15.23 | 0.06 | 15.24 | 0.02 | 10.27 | 0.06 | | |
| | A 3.1 | 15.19 | | | | | | | | |
| | A 3.2 | 15.25 | 15.26 | 0.02 | | | | | | |
| | A 3.2 | 15.27 | | | | | | | | |
| | 3.1 | 25.55 | 25.51 | 0.05 | | | | | | |
| | 3.1 | 25.47 | | | | | | | | |

**Supplementary Table 1.** Exemplary ΔCq calculation for a single assay. Each sample is run in a series of replicates – there are total of three biological replicates (1–3×), each of them is performed in preAMP duplicate (A1.1 and A1.2), and in qPCR replicate (e.g., A1.1, A1.1). Mean Cq is calculated progressively at each replicate level, until ΔCq is obtained for each sample (the "non-preamp – preamp" column). Finally, the $SD_{\Delta Cq}$ is calculated as standard deviation from the three individual ΔCqs (the "preAMP mix" column).

## 3. Quantitative PCR

### 3.1. Low-Throughput qPCR

Reagents

- TATAA SYBR® GrandMaster® mix (TATAA Biocenter, Göteborg, Sweden), cat. no. TA01-625
- 10 µM assay primers of interest (equimolar mixture of forward and reverse primer)

Notes:

- Consider using multichannel pipette for adding the samples and repeat-dispense pipette for distributing the mastermix.
- Every qPCR run should include positive control, negative control (nuclease-free water), gDNA and interplate calibrator (IPC). When bulk sample is used as an IPC, it simultaneously fulfills the role of positive control. Store IPC aliquoted.

Protocol

*Timing: preparation and pipetting of 40 min; thermal protocol of 90 min.*

a. Thaw, vortex and spin all the reaction components before use. Thaw samples on ice and spin briefly. Perform all the pipetting steps on ice.
b. Prepare qPCR mastermix for each assay of interest in a separate DNase-free microtube. When preparing more than one reaction, prepare the qPCR mastermix in 10% surplus. Mix the following components in a DNase-free tube:

| qPCR | Volume Per One qPCR Reaction (µl) | Stock Concentration | Final Concentration |
|---|---|---|---|
| TATAA SYBR® GrandMaster® mix | 5.0 | 2× | 1× |
| Nuclease-free water | 2.6 | – | – |
| Assay primers | 0.4 | 10 µM | 0.4 µM |
| Preamplified cDNA (4× dil.) | 2.0 | – | – |

c. When ready, briefly vortex and spin. Then distribute 8 µl of the qPCR mastermix into qPCR-compatible plate.
d. Add 2 µl 4× diluted preamplified cDNA sample, briefly vortex, spin and incubate:

| Temperature | Time | | Notes |
|---|---|---|---|
| 95 °C | 3 min | | |
| 95 °C | 15 s | 45 cycles | Fluorescence detection |
| 60 °C | 20 s | | |
| 72 °C | 20 s | | |
| 65 °C to 95 °C | Increment = 0.5 °C | | Melting curve analysis |

*Note: Temperature profile can be adjusted to particular assays or mastermixes used.*

e. Data pre-processing steps are listed in 4. Data Pre-Processing section.

3.2. High-Throughput qPCR

Follow-up high-throughput qPCR protocol describes use of BioMark™ HD platform (Fluidigm, South San Francisco, California USA) which allows processing up to 96 samples on 96 assays simultaneously.

Reagents

For qPCR assay plate:

- 2× Assay Loading Reagent (Fluidigm, South San Francisco, California USA), cat. no. 100-7611
- 10 µM assay primers of interest (equimolar mixture of forward and reverse primer)

For qPCR sample plate:

- 20× DNA Binding Dye Sample Loading Reagent (Fluidigm, South San Francisco, California USA), cat .no. 100-7609
- 2× SSoFast EvaGreen Supermix (Bio-Rad, Hercules, California USA), cat. no. 172-5200
- ROX Reference Dye (ThermoFisher Scientific, Waltham, MA USA), cat .no. 12223012

Notes

- The sample list should include control samples, such as IPC, no-template control (nuclease-free water) or gDNA.
- The assay list should include assays monitoring reaction status – spike-in for the technical issues and ValidPrime® (TATAA Biocenter, Göteborg, Sweden, cat. no. A106P25) for genomic background.

- Consider using multichannel pipette for adding the samples and repeat-dispense pipette for distributing the mastermix.

Protocol

*Timing: preparation and pipetting in ~ 90 min, chip priming in 30 min, thermal protocol of 120 min.*

a. Vortex and spin all reagents before use. Thaw samples on ice and spin briefly. Perform all the pipetting steps on ice.
b. Prepare qPCR assay mastermix by combining 2× Assay Loading Reagent with nuclease-free water in a DNase-free tube. For entire plate setups prepare the mastermix in surplus.

| qPCR Assay Mastermix | Volume Per One qPCR Reaction (µl) | Volume Per 96 Wells + Surplus (µl) | Stock Concentration | Final Concentration |
|---|---|---|---|---|
| 2× Assay Loading Reagent | 3.25 | 344.5 | 2× | 1× |
| Nuclease-free water | 0.65 | 68.9 | – | – |
| 10 µM assay primers | 2.60 | – | 10 µM | 4 µM |

c. Shortly vortex, spin and distribute the qPCR assay mastermix by 3.9 µl into new PCR plate.
d. Add 2.6 µl of 10 µM assay primers of interest into individual wells. When added, briefly vortex and spin the prepared qPCR assay plate. Keep on ice.
*Note: Adding 10 µM assay primers on one-by-one basis is a laborious task and it is susceptible to pipetting error. Instead, we recommend preparing a separate PCR plate with individual 10 µM assay primers of interest and pipette these with multichannel pipette.*
e. Fill microfluidics of the 96.96 Dynamic Array IFC chip (Fluidigm, South San Francisco, California USA) with oil and set the IFC Controller (Fluidigm, South San Francisco, California USA) for priming (~30 min procedure). In the meantime, prepare qPCR sample mastermix.
f. Prepare qPCR sample mastermix by combining 20× DNA Binding Dye Sample Loading Reagent, 2× SSoFast EvaGreen Supermix, ROX Reference Dye and nuclease-free water in a DNase-free tube. For entire plate prepare the mastermix in surplus:

| qPCR Sample Mastermix | Volume Per One qPCR Reaction (µl) | Volume Per 96 Wells + Surplus (µl) | Stock Concentration | Final Concentration |
|---|---|---|---|---|
| 20× DNA Binding Dye Sample Loading Reagent | 0.42 | 43.32 | 20× | 1× |
| 2× SSoFast EvaGreen Supermix | 4.17 | 433.16 | 2× | 1× |
| ROX Reference Dye | 0.017 | 1.73 | – | – |
| Nuclease-free water | 0.4 | 41.58 | – | – |
| Preamplified cDNA (4× dil) | 3.33 | – | – | – |

g. Shortly spin, vortex and distribute the qPCR sample mastermix by 5 µl into new PCR plate.
h. Add 3.33 µl of 4× diluted preamplified cDNA sample into individual wells. When added, briefly vortex and spin the prepared qPCR sample mastermix plate. Keep on ice.

i. Pipette the contents of qPCR assay plate and qPCR sample plate into the chip by 5.5 µl and 6 µl, respectively.
*Note: The mastermixes need to be pipetted without introducing bubbles into the chip wells. Dispensing the volume only into pipette's "first position" can help to alleviate the problem. If bubbles are introduced, aspirate them using pipette with narrow tips. Beware of cross-contamination.*

j. Filled chip is set for loading in the IFC Controller. Loaded chip is then placed into BioMark™ HD system (Fluidigm, South San Francisco, California USA) for incubation:

| Temperature | Time | | Notes |
|---|---|---|---|
| 95 °C | 3 min | | |
| 95 °C | 5 s | 35 cycles | Fluorescence detection |
| 60 °C | 15 s | | |
| 72 °C | 20 s | | |
| 65 °C to 95 °C | Increment = 0.5 °C | | Melting curve analysis |

*Note: Temperature profile can be adjusted to particular assays or mastermixes used.*

k. Data pre-processing steps are described in 4. Data Pre-Processing section.

3.3. qPCR Assay Validation

qPCR assays are key determinants of reaction specificity, efficiency and reproducibility. Any assay that is being employed in scRT-qPCR measurements should be screened at minimum for *Overall assay performance* and *Assay efficiency*. Assays targeting low-expressed transcripts should be additively screened for *Limit of detection* and *Limit of quantification*.

a. Prepare new assays. Design at least two assay variants for each targeted transcript, following the rules described in the chapter ***Assay design*** in the main text. Design individual assay variants in different exons to minimize the risk of low performance due to secondary structures, nucleotide composition etc.

*Overall assay performance.*

*Timing: Reverse Transcription in ~60 min in total, low-throughput qPCR in 150+ min in total*

b. Performance of each new assay is validated using cDNA, gDNA and no-template samples. cDNA concentration is adjusted based on the expected target concentration. gDNA concentration mimics low level of gDNA contamination (e.g., ~1 ng per reaction, equivalent of hundreds of genomes).

c. Use protocol for 3.1 Low-Throughput qPCR. Minimum of six samples are processed per each assay variant, cDNA in $n \geq 2$ replicates, gDNA in one replicate and nuclease-free water in $n \geq 3$ replicates. After the run, store the PCR products at 4 °C for further testing.
Note: *Use technical cDNA replicates. This way, assay variability may be estimated based on the total reaction variance.*

d. Proceed with further screening with assays that report: i) no signal or Cq > 40 for nuclease-free water samples, ii) Cq > 35 for gDNA, and iii) standard deviation of Cq for cDNA samples ($SD_{Cq}$) < 0.3. Reaction specificity needs to be verified by the melt curve analysis and correct product size inspects on gel electrophoresis. If more assay variants meet the requirements, select the one reporting lowest Cq for cDNA samples.

*Assay efficiency.*

*Timing: dilution series prepared in ~45 min, qPCR in 150+ min in total*

e. Efficiency of selected assays is determined by dilution series measurement using PCR products from the *Overall assay performance* step as template. Typically, PCR product is 100 000× diluted in post-PCR room and used to prepare the first dilution. In total, the dilution series consists of six points (10-fold dilutions), covering Cq values from 20 to 40. Dilution series is prepared in TE buffer supplied with linear polyacrylamide (LPA) or other molecules serving as a carrier to minimize the binding of molecules to plastic surfaces that would negatively affect signals from samples containing limiting number of molecules.
f. For each assay, the dilution series should be measured minimally in $n \geq 3$ replicates. Set up the measurement according to 3.1 Low-Throughput qPCR protocol.
g. Resulting Cq values are fitted into linear regression model (Cq ~ dilution), from which assay efficiency can be calculated:

$$E = 10^{-1/a} \quad \text{(Equation 1)}$$

where $a$ refers to the slope of the linear fit. Assays selected for scRT-qPCR should record efficiency $E$ between 0.9–1.05 (90–105%) [13,21].

*Limit of detection and limit of quantification.*

*Timing: cleanup and electrophoresis of PCR products in ~4 h in total, dilution series prepared in ~45 min and qPCR in 150+ min.*

h. LoD and LoQ are calculated from a standard curve with known number of amplicons per reaction volume. PCR products from cDNA samples (*Overall assay performance* step) are suited as a template.
i. Clean the PCR products by PCR purification kit of choice and check for correct fragment length by gel or capillary electrophoresis.
j. Knowing size and concentration of cleaned amplicons, one can calculate the concentration of target molecules:

$$\textit{theoretical weight [g/mol]} = (\textit{amplicon.length} \times 607.4) \quad \text{(Equation 2)}$$

$$\textit{number of copies} = (\textit{amount [ng]} \times N_A) / (\textit{theoretical weight} \times 10^9) \quad \text{(Equation 3)}$$

where *amplicon.length* corresponds to number of base pairs in the dsDNA amplicon, *theoretical weight* approximates weight of an amplicon, $N_A$ is Avogadro's number, and *number of copies* is obtained for desired *amount* of amplicons (in nanograms).

k. With copy numbers known, serial dilution of minimum six dilutions spanning the range 100 to 1 copy per qPCR can be prepared. More dilution points in the limiting concentrations are preferred (e.g., 100 – 50 – 25 – 12.5 – 6 – 3 – 1.5 – 0.75 amplicons per qPCR reaction).
l. Prepare the qPCR reactions in replicates $n \geq 6$ per assay, following the 3.1 Low-Throughput qPCR protocol.
m. Although LoD and LoQ can be then determined with great precision [14], their values can be readily approximated: LoD can be determined as the lowest amplicon count for which all qPCR replicates reported specific signal. LoQ can be determined as the lowest amplicon count for which variance among qPCR replicates corresponds to $SD_{Cq} < 0.5$.

## 3.4. Sample Quality Control

Sample quality control is a voluntary screening step with the goal to prevent wasting of reagents on material of poor quality. Typically, non-preamplified single-cell cDNA is used for screening of highly

expressed cell markers (e.g., cell type, cell cycle, precursor). Cells can be measured for up to five markers with the described protocol.

*Timing: Reverse transcription in ~60 min in total, qPCR in ~150+ min in total.*

a. Single-cell RNA is reverse-transcribed into cDNA by RT protocol of choice (1. Reverse Transcription). cDNA is left undiluted.
b. Next, qPCR reactions for marker assays are set up:

| Sample Quality Control (qPCR) | Volume Per One qPCR Reaction (µl) | Stock Concentration | Final Concentration |
|---|---|---|---|
| TATAA SYBR® GrandMaster® mix | 5.0 | 2× | 1× |
| Nuclease-free water | 3.6 | – | – |
| preAMP assay pool | 0.4 | 10 µM | 0.4 µM |
| Non-preamplified cDNA (undiluted) | 1.0 | – | – |

c. Mastermix is then distributed into PCR-compatible plate by 9 µl.
d. 1 µl of undiluted non-preamplified cDNA is then added to the wells.
*Note: Only limited number of assays (up to five) can be screened per cell, as 4 µl undiluted cDNA are needed in the preamplification step.*
e. For details regarding qPCR preparation and incubation, please see 3.1. Low-Throughput qPCR.
f. Based on the marker assays, select for cells that meet selection criteria.

## 4. Data Pre-Processing

a. Firstly, the shape of melting curves is investigated. Distorted shape of the melting peak (widened or double peak) is indicative of unspecific co-amplification [22]. Signals exhibiting substantial contaminations by unspecific products are discarded.
b. If multiple qPCR runs are merged, Cq values need to be interplate calibrated. For each qPCR run, the Cq values are adjusted by margin which the plate's IPC differs from the average IPC across the calibrated runs. For more about IPC sample see main text ***Quantitative PCR – Quality Control***.
c. To identify and remove outlier reactions, absolute Cq values are checked. Reactions reporting: i) Cq > 40 for non-preamplified cDNA, or ii) Cq > 28 for preamplified material are discarded [23,24].
d. In the next step, Cq values are transformed to relative quantities for every assay separately:

$$RQ = 2^{Cq_{MAX} - Cq} \quad \text{(Equation 4)}$$

where $Cq_{MAX}$ is the highest measured Cq per assay per given dataset.

e. Missing values are accounted for by imputation. For data imputation please see the main text (**Data analysis – Data pre-processing**).
f. Lastly, RQ values are $\log_2$-transformed.
g. For follow-up analysis steps, see [21,23,24].

## References


1. Androvic, P.; Kirdajova, D.; Tureckova, J.; Zucha, D.; Rohlova, E.; Abaffy, P.; Kriska, J.; Valny, M.; Anderova, M.; Kubista, M.; et al. Decoding the Transcriptional Response to Ischemic Stroke in Young and Aged Mouse Brain. *Cell Rep.* **2020**, *31*, 107777, doi:10.1016/j.celrep.2020.107777.
2. Kolenicova, D.; Tureckova, J.; Pukajova, B.; Harantova, L.; Kriska, J.; Kirdajova, D.; Vorisek, I.; Kamenicka, M.; Valihrach, L.; Androvic, P.; et al. High potassium exposure reveals the altered ability of astrocytes to regulate their volume in the aged hippocampus of GFAP/EGFP mice. *Neurobiol. Aging* **2020**, *86*, 162–181, doi:10.1016/j.neurobiolaging.2019.10.009.
3. Pivonkova, H.; Hermanova, Z.; Kirdajova, D.; Awadova, T.; Malinsky, J.; Valihrach, L.; Zucha, D.; Kubista, M.; Galisova, A.; Jirak, D.; et al. The Contribution of TRPV4 Channels to Astrocyte Volume Regulation and Brain Edema Formation. *Neuroscience* **2018**, *394*, 127–143, doi:10.1016/j.neuroscience.2018.10.028.
4. Valny, M.; Honsa, P.; Waloschkova, E.; Matuskova, H.; Kriska, J.; Kirdajova, D.; Androvic, P.; Valihrach, L.; Kubista, M.; Anderova, M. A single-cell analysis reveals multiple roles of oligodendroglial lineage cells during post-ischemic regeneration. *Glia* **2018**, *66*, 1068–1081, doi:10.1002/glia.23301.
5. Dzamba, D.; Valihrach, L.; Kubista, M.; Anderova, M. The correlation between expression profiles measured in single cells and in traditional bulk samples. *Sci. Rep.* **2016**, *6*, 37022, doi:10.1038/srep37022.
6. Honsa, P.; Valny, M.; Kriska, J.; Matuskova, H.; Harantova, L.; Kirdajova, D.; Valihrach, L.; Androvic, P.; Kubista, M.; Anderova, M. Generation of reactive astrocytes from NG2 cells is regulated by sonic hedgehog. *Glia* **2016**, *64*, 1518–1531, doi:10.1002/glia.23019.
7. Dzamba, D.; Honsa, P.; Valny, M.; Kriska, J.; Valihrach, L.; Novosadova, V.; Kubista, M.; Anderova, M. Quantitative Analysis of Glutamate Receptors in Glial Cells from the Cortex of GFAP/EGFP Mice Following Ischemic Injury: Focus on NMDA Receptors. *Cell. Mol. Neurobiol.* **2015**, *35*, 1187–1202, doi:10.1007/s10571-015-0212-8.
8. Anderova, M.; Benesova, J.; Mikesova, M.; Dzamba, D.; Honsa, P.; Kriska, J.; Butenko, O.; Novosadova, V.; Valihrach, L.; Kubista, M.; et al. Altered Astrocytic Swelling in the Cortex of $\alpha$-Syntrophin-Negative GFAP/EGFP Mice. *PLoS One* **2014**, *9*, e113444, doi:10.1371/journal.pone.0113444.
9. Benesova, J.; Rusnakova, V.; Honsa, P.; Pivonkova, H.; Dzamba, D.; Kubista, M.; Anderova, M. Distinct Expression/Function of Potassium and Chloride Channels Contributes to the Diverse Volume Regulation in Cortical Astrocytes of GFAP/EGFP Mice. *PLoS One* **2012**, *7*, e29725, doi:10.1371/journal.pone.0029725.
10. Rusnakova, V.; Honsa, P.; Dzamba, D.; Ståhlberg, A.; Kubista, M.; Anderova, M. Heterogeneity of astrocytes: from development to injury - single cell gene expression. *PLoS One* **2013**, *8*, e69734, doi:10.1371/journal.pone.0069734.
11. Zucha, D.; Androvic, P.; Kubista, M.; Valihrach, L. Performance Comparison of Reverse Transcriptases for Single-Cell Studies. *Clin. Chem.* **2020**, *66*, 217–228, doi:10.1373/clinchem.2019.307835.
12. Valihrach, L.; Androvic, P.; Kubista, M. Platforms for Single-Cell Collection and Analysis. *Int. J. Mol. Sci.* **2018**, *19*, 807, doi:10.3390/ijms19030807.
13. Ståhlberg, A.; Kubista, M. Technical aspects and recommendations for single-cell qPCR. *Mol. Aspects Med.* **2018**, *59*, 28–35, doi:10.1016/j.mam.2017.07.004.
14. Forootan, A.; Sjöback, R.; Björkman, J.; Sjögreen, B.; Linz, L.; Kubista, M. Methods to determine limit of detection and limit of quantification in quantitative real-time PCR (qPCR). *Biomol. Detect. Quantif.* **2017**, *12*, 1–6, doi:10.1016/J.BDQ.2017.04.001.
15. Svec, D.; Tichopad, A.; Novosadova, V.; Pfaffl, M.W.; Kubista, M. How good is a PCR efficiency estimate: Recommendations for precise and robust qPCR efficiency assessments. *Biomol. Detect. Quantif.* **2015**, *3*, 9–16, doi:10.1016/j.bdq.2015.01.005.
16. Andersson, D.; Akrap, N.; Svec, D.; Godfrey, T.E.; Kubista, M.; Landberg, G.; Ståhlberg, A. Properties of targeted preamplification in DNA and cDNA quantification. *Expert Rev. Mol. Diagn.* **2015**, *15*, 1085–1100, doi:10.1586/14737159.2015.1057124.
17. Svec, D.; Andersson, D.; Pekny, M.; Sjöback, R.; Kubista, M.; Ståhlberg, A. Direct Cell Lysis for Single-Cell Gene Expression Profiling. *Front. Oncol.* **2013**, *3*, 1–11, doi:10.3389/fonc.2013.00274.
18. Bustin, S.A.; Benes, V.; Garson, J.; Hellemans, J.; Huggett, J.; Kubista, M.; Mueller, R.; Nolan, T.; Pfaffl, M.W.; Shipley, G.; et al. The need for transparency and good practices in the qPCR literature. *Nat. Methods* 2013, *10*, 1063–1067.
19. Korenková, V.; Scott, J.; Novosadová, V.; Jindřichová, M.; Langerová, L.; Švec, D.; Šídová, M.; Sjöback, R. Pre-amplification in the context of high-throughput qPCR gene expression experiment. *BMC Mol. Biol.* **2015**, *16*, 5, doi:10.1186/s12867-015-0033-9.
20. Bagnoli, J.W.; Ziegenhain, C.; Janjic, A.; Wange, L.E.; Vieth, B.; Parekh, S.; Geuder, J.; Hellmann, I.; Enard, W. Sensitive and powerful single-cell RNA sequencing using mcSCRB-seq. *Nat. Commun.* **2018**, *9*, 2937, doi:10.1038/s41467-018-05347-6.

21. Ståhlberg, A.; Rusnakova, V.; Kubista, M. The added value of single-cell gene expression profiling. *Brief. Funct. Genomics* **2013**, *12*, 81–89, doi:10.1093/bfgp/elt001.
22. Bustin, S.; Huggett, J. qPCR primer design revisited. *Biomol. Detect. Quantif.* 2017, *14*, 19–28.
23. Ståhlberg, A.; Rusnakova, V.; Forootan, A.; Anderova, M.; Kubista, M. RT-qPCR work-flow for single-cell data analysis. **2013**, *59*, 80–88, doi:10.1016/j.ymeth.2012.09.007.
24. Bergkvist, A.; Rusnakova, V.; Sindelka, R.; Garda, J.M.A.; Sjögreen, B.; Lindh, D.; Forootan, A.; Kubista, M. Gene expression profiling - Clusters of possibilities. *Methods* **2010**, *50*, 323–335, doi:10.1016/j.ymeth.2010.01.009.
